# Impact of Care Bundles Including Preoperative Skin Preparation and Vaginal Cleaning on Preventing Surgical Site Infections in Caesarean Deliveries

**DOI:** 10.7759/cureus.77788

**Published:** 2025-01-21

**Authors:** Shivangi Ghildiyal, Savita Somalwar, Anuja Bhalerao

**Affiliations:** 1 Department of Obstetrics and Gynaecology, Narendra Kumar Prasadrao (NKP) Salve Institute of Medical Sciences and Research Centre, Nagpur, IND

**Keywords:** caesarean section, preoperative skin preparation, surgical bundle, surgical site infections, vaginal cleaning

## Abstract

Caesarean section (CS) is one of the most frequent surgical procedures performed. Consequently, in obstetrical situations, particularly in emergency CS, post-CS surgical site infections (SSIs) are frequent. SSIs are a major contributor to infectious morbidity which occurs after any invasive surgical treatment at the incision site. Care bundles are a group of coordinated, methodical measures used to reduce SSI that may also enhance patient outcomes along with preoperative preparation of the skin and cleaning of the vagina. Hence, the present scoping review aims to investigate the impact of surgical care bundles, preoperative skin preparation, and vaginal cleaning on SSI prevention in women undergoing CS. A literature search was conducted between 2019 and 2024 utilizing electronic databases such as Science Direct, PubMed, and Google Scholar. The keywords used consisted of, “caesarean section”, AND “surgical site infection”, OR “pre-operative skin preparation”, OR “vaginal cleaning”, OR “surgical bundle” to include relevant articles in the review. Based on the selection criteria, seven studies were included. The results of the included trials reported a notable decrease in post-CS SSI with the use of a bundled method including vaginal cleaning and preoperative skin preparations; nevertheless, larger investigations are required to confirm the precise involvement. Hence, the utilization of evidence-based information to develop appropriate surgical bundles to decrease the rates of SSIs after CS is highly recommended.

## Introduction and background

Despite the resulting implications, the past few decades have witnessed a remarkable global rise in caesarean section (CS) surgeries. Following CS, the major cause of morbidity is postoperative complications for both the mother and the fetus [1]. Surgical site infections (SSI) are one major contributor to infectious morbidity which occurs after any invasive surgical treatment at the incision site. Consequently, in obstetrical situations, particularly in emergency CS, post-CS SSI is frequent [2]. As these infections affect women's physical and mental health, every effort should be made to enhance the quality of life of the woman through prevention.

While the frequency of SSI following CS varies from clinic to clinic, it typically occurs in the first week of postpartum and falls between the range of 2% and 19% [3]. The majority of infections occur through an ascending pathway by the cervicovaginal flora [4]. The risk of infection is increased by bacterial vaginosis, prolonged rupture of the premature membranes, prolonged labour, and repeated examinations of the vagina [5,6]. Even with the preoperative prophylactic use of broad-spectrum antibiotics, postoperative infection cannot be eradicated today. Consequently, preoperative vaginal cleansing with povidone-iodine is a safe, affordable, and simple technique for the eradication of fungus and bacteria and decreases postoperative infections in addition to antibiotic prophylaxis [7,8]. Care bundles are a group of coordinated, methodical measures used to reduce SSI that may also enhance patient outcomes. The success of a bundle hinges on the constant and persistent implication of all measures, even though individual interventions may differ throughout bundles. The optimal care bundle consists of hair clipping, glycaemic control, and antibiotic therapy. Other components may be included synergistically [1]. Therefore, this scoping review aims to investigate the impact of surgical bundles, preoperative skin preparation, and vaginal cleaning for SSI prevention in women undergoing CS.

## Review

Review

Preferred Reporting Items for Systematic Reviews and Meta-Analyses for Scoping Review (PRISMA-ScR) guidelines were followed while this scoping review was conducted [9].

Data sources and search strategy

A literature search was conducted for the articles published between 2019 to 2024 with the latest on 5th August 2024, with the keywords “caesarean section”, AND “surgical site infection”, OR “pre-operative skin preparation”, OR “vaginal cleaning”, OR “surgical bundle” on electronic databases that included Google Scholar, Science Direct, and PubMed.

Study screening and selection

The inclusion criteria for the screening process included studies with women undergoing CS in which the effect of the surgical bundle with preoperative skin preparation and vaginal cleaning on the prevention of SSIs was observed, studies published between 2019 and 2024, cross-sectional studies, randomized controlled trials, retrospective observational studies, and studies full-text availability and published in the English language. Study designs that, however, included case reports, review articles, book chapters, commentary, editorials, guidelines, and letters to the editor were not included. Other exclusions included studies that were not published in English with full-text non-availability, and studies that provided inadequate knowledge based on the context.

Two reviewers independently assessed each article to determine the applicability of the studies. First, titles and abstracts were reviewed to eliminate duplicate articles. Second, additional screening was performed on the chosen articles to eliminate those that did not follow the eligibility requirements. Lastly, to assess eligibility, the full text of the selected articles was used for screening. Any disagreements or conflicts were resolved through discussions and consensus among the reviewers.

Data extraction

The data retrieved independently by the reviewers consisted of the purpose and objectives of the investigations, the sample size, the study design, the methodology, the results, the conclusion, and the quality assessment. Each retrieved information was examined separately before inclusion in the review.

Quality assessment

Using the Mixed Methods Appraisal Tool (MMAT) [10], the methodological quality of the included studies was evaluated. Studies involving non-randomized trials, using mixed techniques, randomized controlled trials, cross-sectional, and qualitative, are frequently evaluated with this tool [11,12]. The quality was graded as high, low, or moderate.

Data synthesis

The results of the studies were combined using the critical narrative technique. Figures, text, and tables are used in the narrative synthesis to support and enumerate the study findings [11]. For the review, a critical perspective was provided by higher methodological quality research, potential biases, limitations, and other issues during the data analysis. As the number of relevant research was limited, statistical synthesis or a meta-analysis was not performed. There is a considerable amount of heterogeneity in the included studies since different research procedures and outcome measures were taken into account.

Results

**Figure 1:**
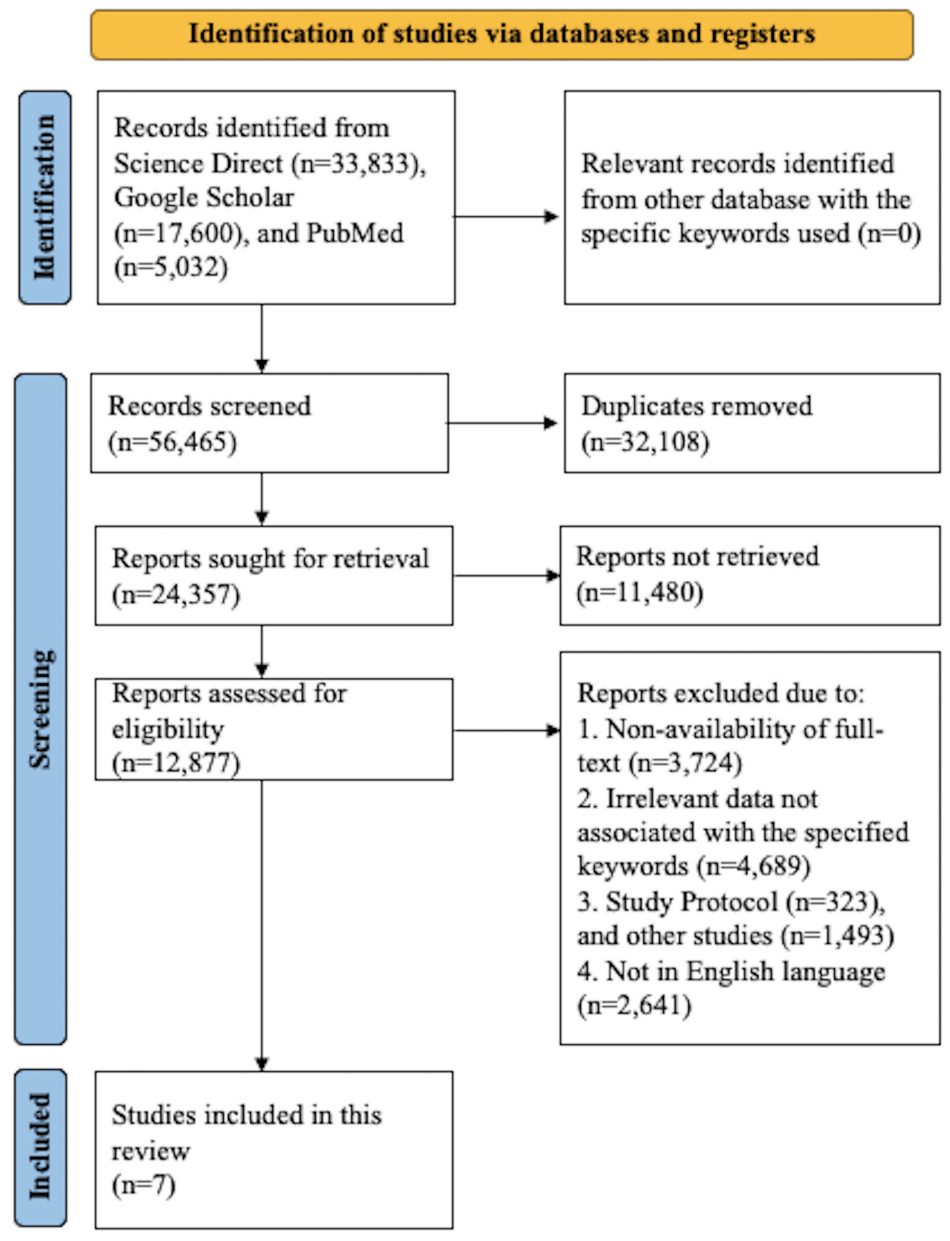
PRISMA flowchart illustrating search strategy

The search strategy is outlined in Figure 1. Overall, 56,465 articles were screened which included 5,032 from PubMed, 33,833 studies from Science Direct, and 17,600 articles from the Google Scholar databases. After 32,108 duplicate articles were removed, 24,357 were left and put through a retrieval evaluation from which 11,480 were not retrieved. The next step involved screening 12,877 articles for eligibility; of these, 4,689 reported irrelevant data based on the keywords; 3,724 indicated full-text non-availability; 1,816 were other studies; and 2,641 were not in English and were thus excluded. Hence, a total of seven studies were included.

Moreover, the extracted data from the included studies is described in Table 1.

**Table 1:** Summary of the extracted data CS: caesarean section; SSI: surgical site infections; CRP: C-reactive protein; VAS: visual analague scores; CHG: chlorhexidine gluconate

Sr. No.	Author and year	Aim and Objectives	Study design	Sample size	Methodology	Results	Conclusion	Quality assessment
1.	Nagori et al. (2021) [1]	To evaluate the contribution of vaginal cleaning and the use of surgical bundles to the prevention of postoperative infectious complications before CS.	Prospective randomized interventional study	216	There were two groups of patients. Women in group 1 who were having emergency CS were given a 7-day postoperative prophylactic antibiotic regimen along with standard preoperative preparation that included the use of Savlon-povidone iodine as a skin antiseptic. Group 2 had vaginal cleansing and a surgical bundle, but the postoperative prophylactic antibiotic regimen was shortened to three days.	In comparison to Group 1 the incidence of SSIs was lowest in Group 2 and the results were significant statistically.	The incidence of SSIs following emergency CS has decreased with the use of a bundled method including vaginal cleaning; nevertheless, larger investigations are required to confirm the precise involvement.	High
2.	Kanza et al. (2021) [6]	The purpose of the current study was to assess the impact of vaginal cleaning with povidone-iodine or saline solution before elective CS on postoperative infection, and postpartum maternal morbidity.	Randomized controlled trial	180	Three groups consisting of 180 primiparae who were waiting for elective CS were formed: Group 1 (vaginal cleansing with saline solution for 30 seconds; n = 60); Group 2 (vaginal cleansing with povidone-iodine for 30 seconds; n = 60); Group 3 (control group, n = 60).	In the saline solution group, the median postoperative CRP level was 26.5 mg/dl; in the control group, it was 62.3 mg/dl, and in the povidone-iodine group, it was 59.5 mg/dl. The corresponding median VAS scores were 3, 4, and 4, and 1.7%, 3.4%, and 10% of patients respectively had a temperature higher than 38°C.	Before CS, vaginal cleansing with povidone-iodine or normal saline solution decreased postoperative fever, pain, and CRP levels. Endometritis and post-CS wound site infections were clinically and significantly reduced when the vagina was cleaned before a CS.	High
3.	Scholz et al. (2021) [13]	To examine clinical outcomes before and after implementation of the SSI bundle.	Clinical trial	3,637	Following an increase in CS-associated SSI, the introduction of the preventative bundle was associated with the institution's transition from povidone-iodine to CHG/isopropanol in preoperative scrub. The bundle comprised surgery prep retraining, a full body preoperative wash with 4% CHG cloths, wound care instruction for patients, and retraining for surgeons doing hand scrubs. Four epochs were identified over seven years, involving patients delivered via CS at 24 weeks gestation: baseline (18 months using povidone-iodine); CHG scrub (18 months after switching from skin prep to CHG); bundle implementation (24 months); and maintenance (24 months following implementation).	In epochs, 1–4, 667, 796, 1098, and 1076 women respectively, were included. The switch to CHG from povidone-iodine resulted in an increase in SSI from 8.4–13.3%. This rate dropped to 4.5% after the intervention (maintenance epoch). This can be explained by a decline in wound infection (rates were 6.9, 12.9, and 3.5% respectively in the three epochs prior), with endometritis remaining unchanged.	A decrease in SSI post-CS was associated with the implementation of a preventative bundle. Beyond the recommended preoperative dosage, no additional antibiotics were used for this improvement.	High
4.	Bolte et al. (2020) [14]	To develop, practice, and evaluate a bundled intervention for Caesarean Infection Prevention ("CIP") in a high-risk population to reduce SSI rates post-CS.	Pilot pre-post-intervention study	710	Women who experienced a post-CS SSI were identified. The intervention was developed with input from a thorough literature evaluation and went into effect in December 2017. For the next 12 months, information on women undergoing CS was collected.	346 women were in the pre-intervention group and 364 women were in the post-intervention group. The most impact was seen in obese class II and III patients, where SSI rates considerably decreased post-CS.	Post-CS, SSIs were effectively reduced by the "CIP" bundle.	High
5.	Davidson et al. (2020) [15]	To evaluate the rates of SSI both before and after an SSI care bundle was implemented in CS.	Retrospective cohort study	4,014	Women were categorized into pre- and post-bundle groups. The rate of SSI was evaluated.	After the SSI bundle was implemented, the mean in the pre-bundle group was reduced from 2.44 to 1.1 representing a 221% decrease in the SSI rate. Additionally, there was good compliance with the bundle elements	SSI rates in CS were shown to have significantly decreased after the implementation of the infection prevention bundle.	High
6.	Borhanzehi et al. (2024) [16]	To investigate the impact of care bundle on SSI prevention post-CS.	Randomized clinical trial	60	Overall, 60 pregnant women were selected and divided into two groups of 30 each. In the control group, patients received standard treatment, while in the intervention group, patients received a care bundle for infection prevention for pre-, intra, and postoperative care. The surgical incision was examined at 24 hours, 10th, and 30th days following surgery to confirm or rule out wound infection. REEDA Scale and the wound assessment checklist were used for this purpose. An SSI was verified by a score higher than 6.	Within the control group, the mean REEDA scores were 3.64 ± 5.26, 1.7 ± 3.07, and 0.08 ± 0.3 for patients after surgery at 24 hours, 10th and 30th days respectively. whereas, in the intervention group were 2.52 ± 4.6, 1.3 ± 2.7, and 0, respectively with statistically insignificant differences. Furthermore, there was a statistically insignificant difference in the number of patients exhibiting signs and symptoms of SSI between the control group (n = 5) and the intervention group (n = 2).	The benefits of care bundles in preventing SSIs cannot be denied, even though the results indicated that they were ineffective in preventing CS wound infections. As a result, the care bundle's components should be closely inspected, and any possible problems should be further investigated in follow-up research. This is especially true of intraoperative interventions, which require close coordination between the operating room and surgical nurses and technicians.	High
7.	Kawakita et al. (2019) [17]	To investigate resident-driven quality initiatives in association with SSIs in CS.	Quasi-experimental, pre-intervention and post-intervention study	1,624	An evidence-based surgical bundle led by residents was implemented. The bundle included using hair clippers, skin preparation with chlorhexidine alcohol, vaginal cleaning with povidone-iodine, suture skin closure, removal of dressings after 24 to 48 hours, and placental removal by umbilical cord traction if the wound was thicker than 2 cm. The main result was SSIs that occurred up to six weeks after delivery.	1,100 women in the postimplementation were matched to 1,100 women in the preimplementation. Compared to the pre-implementation (4.5%), and the post-implementation period (2.2%) a significantly lower SSI rate was observed in the unmatched cohort. After matching, the decline in the SSI rate (1.9% versus 4.1%) remained statistically significant.	A significant decrease in the CS SSIs was observed following the implementation of a surgical bundle.	High

Discussion

To avoid SSI in women undergoing CS, the current scoping review evaluated the impact of surgical bundles with preoperative skin preparation and vaginal cleansing. Due to variations in population characteristics, preoperative procedures, related risk factors, and sufficient post-operative surveillance for other sources of infections and SSI, post-operative infectious morbidity varies in incidence. The use of surgical bundles has the greatest impact on SSI incidence of all known techniques. The WHO advises using a single dosage of prophylactic antibiotic as part of a care bundle [16].

The SSI incidence was reportedly reduced in patients who received a care bundle because patients and medical personnel, including doctors, were provided knowledge about easy ways to prevent SSIs. This was also linked to better patient compliance with basic instructions like cleaning their scalp, having a bath before surgery, and having access to facilities. They claimed that the anaesthesia team may potentially lessen the incidence of SSIs by administering preventative antibiotic injections rather than the surgical department's nursing personnel [17]. Similarly, a meta-analysis found that to reduce the risk of SSI these bundles are potential interventions in women undergoing CS because evidence-based care bundles are a collection of therapies that are more successful when used collectively than when used separately [18]. Additionally, Zajac et al. verified that SSI in post-CS patients is decreased by a bundled care strategy [19].

Among the research that was included in the SR, a study presented by Borhanzehi et al. found no evidence of the care bundle's efficacy in preventing CS wound infections [15]. Similar to this, the efficacy of a care bundle using a variety of data was assessed by Jurt et al., including nine intraoperative items, and discovered that because medical staff did not follow individual measures due to inadequate compliance, the care bundle had no influence on the incidence of SSIs [20]. Furthermore, no impact of the bundled therapies was observed on the incidence of SSIs in patients with colorectal surgery, according to a study by Anthony et al. [21]. However, it is undeniable, that the care bundles have a positive impact in preventing or reducing postoperative infections [22], since numerous research have demonstrated the benefits of the SSI preventive care bundle [17-19,23,24].

Strengths and limitations

These studies included were of high quality demonstrating the effectiveness of surgical bundles with preoperative skin preparation and vaginal cleaning for SSIs prevention in women undergoing CS. Certain limitations of the review included the fact that there was no meta-analysis reporting due to the varying methodological section of the studies, and limited studies were included as a result of the imposed selection criteria. Furthermore, the studies in databases other than those considered and published in other languages except English were excluded which may have restricted the quantity of pertinent publications.

## Conclusions

SSIs place a burden on the health care system and SSIs occurring after CS are complicated clinical scenarios that result from a variety of circumstances, including the perioperative management, and patient demographics. The present study highlighted that the combination of the evidence-based components into bundles has been shown to lower SSI post-CS rates. Hence, the utilization of evidence-based information to develop appropriate surgical bundles to decrease the rates of SSIs after CS should be highly recommended.
